# Feasibility Randomized Controlled Trial of ImpulsePal: Smartphone App–Based Weight Management Intervention to Reduce Impulsive Eating in Overweight Adults

**DOI:** 10.2196/11586

**Published:** 2019-04-30

**Authors:** Samantha B van Beurden, Jane R Smith, Natalia S Lawrence, Charles Abraham, Colin J Greaves

**Affiliations:** 1 College of Medicine and Health University of Exeter Exeter United Kingdom; 2 Psychology University of Exeter Exeter United Kingdom; 3 School of Psychological Sciences University of Melbourne Melbourne Australia; 4 School of Sport, Exercise and Rehabilitation Sciences University of Birmingham Birmingham United Kingdom

**Keywords:** weight loss, mHealth, digital behavior change, obesity, dual-process

## Abstract

**Background:**

ImpulsePal is a theory-driven (dual-process), evidence-informed, and person-centered smartphone app intervention designed to help people manage impulsive processes that prompt unhealthy eating to facilitate dietary change and weight loss.

**Objective:**

The aims of this study were to (1) assess the feasibility of trial procedures for evaluation of the ImpulsePal intervention, (2) estimate standard deviations of outcomes, and (3) assess usability of, and satisfaction with, ImpulsePal.

**Methods:**

We conducted an individually randomized parallel two-arm nonblinded feasibility trial. The eligibility criteria included being aged ≥16 years, having a body mass index of ≥25 kg/m^2^, and having access to an Android-based device. Weight was measured (as the proposed primary outcome for a full-scale trial) at baseline, 1 month, and 3 months of follow-up. Participants were randomized in a 2:1 allocation ratio to the ImpulsePal intervention or a waiting list control group. A nested action-research study allowed for data-driven refinement of the intervention across 2 cycles of feedback.

**Results:**

We screened 179 participants for eligibility, and 58 were randomized to the intervention group and 30 to the control group. Data were available for 74 (84%, 74/88) participants at 1 month and 67 (76%, 67/88) participants at 3 months. The intervention group (n=43) lost 1.03 kg (95% CI 0.33 to 1.74) more than controls (n=26) at 1 month and 1.01 kg (95% CI −0.45 to 2.47) more than controls (n=43 and n=24, respectively) at 3 months. Feedback suggested changes to intervention design were required to (1) improve receipt and understanding of instructions and (2) facilitate further engagement with the app and its strategies.

**Conclusions:**

The evaluation methods and delivery of the ImpulsePal app intervention are feasible, and the trial procedures, measures, and intervention are acceptable and satisfactory to the participants.

**Trial Registration:**

International Standard Randomized Controlled Trial Number (ISRCTN): 14886370; http://www.isrctn.com/ISRCTN14886370 (Archived by WebCite at http://www.webcitation.org/76WcEpZ51)

## Introduction

Obesity continues to be a major public health challenge. In the United Kingdom alone, in line with current trends in obesity prevalence, the economic burden of obesity and related health conditions on the National Health Service (NHS) and United Kingdom society is predicted to reach £49.9 billion/year by 2050 [1]. Given this, the development and implementation of cost-effective, scalable weight management interventions is imperative.

Systematic reviews and meta-analyses suggest that behavioral weight management interventions can result in significantly greater weight loss compared with controls (2 kg or more) [2-4]. However, people often struggle to lose or maintain weight, despite their strong intentions to do so [5,6]. This is thought to be due to, at least in part, people’s tendency to make food choices impulsively with little conscious awareness [7,8]. Traditional weight loss interventions focus on conscious, reflective processes, such as planning, monitoring progress, and problem-solving. However, impulsive processes (eg, automatic, habitual, or mindless snacking) are able to undermine conscious reflective processes and are considered to be a major barrier to successful behavior regulation [9-12]. Researchers in the field increasingly recognize the need for interventions that target impulsive, as well as reflective, processes to facilitate health behavior change [12]. This has resulted in the development and evaluation of a range of impulse management techniques with some showing promise in terms of changing eating-related outcomes such as snack intake, craving strength, and body weight [13].

Impulsive processes are triggered by situational cues (eg, [10,11,14]) and individuals may therefore benefit from in-the-moment (or just-in-time) support to modify or otherwise manage such processes for successful behavior change. In 2016, the UK user base for smartphones reached 18% of the population (91% among those aged 18 to 44 years) [15]. Smartphone use continues to permeate daily life with people carrying their phones with them most of the time and looking at them frequently throughout the day [16-18]. Therefore, smartphone apps provide a useful platform for such intervention. Meta-analyses suggest modest effectiveness of mobile health (mHealth) apps targeting weight loss [19,20]. However, reviews of weight loss mHealth apps show that such apps incorporate few theory- and evidence-based features, primarily relying on reflective behavior change techniques such as goal setting and self-monitoring [21,22], rather than techniques specifically supporting impulse management [13].

This study has presented data from a feasibility randomized controlled trial (RCT) of a smartphone app–based weight management intervention, ImpulsePal, that was developed to support dietary behavior change by helping people learn how to modify impulsively regulated eating of unhealthy foods, using evidence-based strategies that explicitly target impulsive processes identified in a recent systematic review [13]. This study encompassed the second stage of the Medical Research Council framework for complex interventions [23] and was designed to (1) inform the planning of a fully powered trial to determine the clinical effectiveness of the intervention in overweight adults and (2) inform the refinement of the intervention in close collaboration with its intended users. Data pertaining to the nested mixed-methods process evaluation are reported elsewhere [24].

The objectives for this feasibility trial were to

1. Assess feasibility of the trial procedures, including rates of recruitment, data collection methods, and retention.
2. Obtain estimates of the SDs of continuous outcome measures to inform sample size calculations for a full-scale trial.
3. Assess the usability of, and satisfaction with, the ImpulsePal intervention and trial methods and procedures.

## Methods

This study has been reported in accordance with the Consolidated Standards for Reporting Trials (CONSORT) [25] recommendations specifically for reporting of pilot RCTs and Template for Intervention Description and Replication (TIDieR) recommendations on reporting of behavior change interventions [26].

### Study Design and Setting

This was a parallel randomized controlled feasibility study with nested quantitative and qualitative process evaluation. Participants were randomized in a 2:1 ratio to the intervention or a waiting list control arm to maximize data on engagement with the intervention. This study incorporated a nested Action Research (AR) study [27,28], with 2 cycles of intervention delivery and user feedback. Refinements were made to intervention content at the end of each cycle, informed by qualitative feedback from participants. Data collection primarily took place at the University of Exeter Medical School. However, home visits were offered to those who were not able to attend study visits at the university. The intervention development and process evaluation are reported in detail elsewhere [24,29], and combined data from both cycles are reported here. This study was approved by the UK NHS National Research Ethics Services Committee South West—Exeter (Ref: 15/SW/0181).

### Participants

Participants were recruited between September 2015 and March 2016 for Cycle 1 and October 2016 and April 2017 for Cycle 2 in the county of Devon in the United Kingdom.

#### Eligibility Criteria

People were eligible to take part if they (1) were aged at least 16 years, (2) had a body mass index (BMI) of 25 kg/m^2^ or more, (3) owned an Android-based smartphone, and (4) lived within a 45-min travelling distance of Exeter, United Kingdom (Devon’s capital city). Exclusion criteria included (1) pregnancy within the last 6 months or planned pregnancy during the study period, (2) not speaking or understanding written English, (3) participation in concurrent weight-related interventional research (though participants could be accessing weight loss services outside of the research), and (4) currently receiving treatment for an eating disorder. Our original protocol required a minimum BMI of 30 kg/m^2^ (and 27.5 for specific ethnicities) but we reduced this to 25 kg/m^2^ to facilitate recruitment and capture a broader range of experiences with the intervention.

#### Identification and Recruitment Routes

At the time the study commenced, weight management services operating in Devon were receiving referrals from General Practitioners or other NHS health professionals. Such referrals were directed to Health Promotion Devon (HPD), a lifestyle hub that helped individuals to select a weight management program from a range of group and one-to-one options. Once a week, a staff member of the HPD referral hub ran a database search to generate a list of people who met the study inclusion criteria and checked for any recorded exclusion criteria (ie, pregnancy and referral to concurrent interventional research). Where appropriate, a study invitation on the HPD letterhead was sent out with the Participant Information Sheet, a reply slip, and a freepost envelope addressed to the researcher (SvB). To allow estimation of the representativeness of the sample recruited in relation to the eligible population, anonymized data including age, gender, (preservice) BMI, and postcode for all individuals who were invited to take part by HPD were requested.

In our original protocol, we stated that we would recruit solely through the local Tier 2 (referral to face-to-face lifestyle intervention) weight management service. However, to increase our recruitment rate and because the HPD service was withdrawn after commencement of the study, additional recruitment routes were added. These included (1) displaying study posters in 3 local GP surgeries, 3 local gym facilities, and 2 local Web-based community noticeboards; (2) offering study flyers to individuals referred to local Tier 3 (hospital-based) weight management services in Devon; (3) inserting a study advert in the university’s newsletter; and (4) placing 2 separate adverts in the Exeter 10,000 project’s (ExTend) yearly newsletter. All adverts, posters, and flyers informed potential participants that (1) the study involved a smartphone app for weight management and (2) a draw-based incentive of a £50 shopping voucher was offered for participation and included the primary investigator’s (SvB) contact details for seeking further information about the study.

### Procedures

#### Telephone Screening and Consent

The people who expressed interest in the study by directly contacting the researcher (SvB) or returning a reply slip were contacted by telephone. The researcher provided further information, addressed any questions about the study, and screened verbally for eligibility. Those who were eligible but declined to participate were invited to give reasons but were not obliged to do so.

#### Consent and Assessments

Potential participants who were eligible and provided oral consent to take part were invited to attend a baseline assessment visit. A baseline invitation pack was sent with information about the visit, and a baseline questionnaire was sent for completion in advance. At the baseline visit, after obtaining written consent, the researcher (1) asked for the questionnaire and checked for completeness and understanding, (2) took other baseline measurements, and (3) randomized the participant to either the intervention or control group. Participants randomized to the intervention group (see below) were provided with instructions for downloading and installing the ImpulsePal app and an anonymized username and password. Follow-up assessments were carried out in the same way at 1 month and 3 months post baseline, although semistructured interviews were conducted at the 1-month follow-up assessment with a subsample of the intervention group only, as part of the process evaluation.

#### Randomization

Participants were allocated in a 2 (intervention) to 1 (control) ratio using a centralized Web-based randomization service [30]. The allocation sequence was stratified in an attempt to achieve balance across the groups in terms of gender, age group (16 to 24, 25 to 35, 36 to 54, and 55+ years), and BMI categories (<35, 35 to 40, >40 kg/m^2^). Block randomization was used, with a block size of 6, to ensure minimal variation from the desired 2:1 ratio. Following entry of a unique participant number and the participant’s gender, age, and BMI, the participant’s allocation code was generated. Neither the participant nor the researcher was aware of group allocation until this point. The same researcher (SvB) enrolled participants and assigned participants to the study arms.

### Intervention

The ImpulsePal intervention was developed using Intervention Mapping methods [31] to (1) support the reduction of unplanned and unhealthy snacking, drinking, and overeating for weight management in people who are overweight, (2) include components for which there was promising evidence that they could modify or otherwise assist in managing impulsive processes related to unhealthy eating, and (3) have the potential for delivery on a large scale. Drawing on dual-process approaches (eg, Reflective Impulsive Model [10]), the intervention contains techniques that help manage the impulsive processes by either preventing their initiation or modifying the direction or strength of the triggered impulse (impulse-focused techniques) or using cognitive resources in identifying and suppressing the impulsively activated behavioral schemas (reflective techniques) [13]. As well as building on our systematic review of techniques to modify impulsive processes [13], the development process involved extensive consultation with service users and behavior change experts.

The intervention is described in the Multimedia Appendix 1, and fuller details of the intervention and its development are described elsewhere [24,29]. Briefly, ImpulsePal is a self-delivered smartphone app that aims to help people modify or manage impulsive processes to facilitate dietary changes (such as reductions in snack consumption). Table 1 presents the key components of ImpulsePal comprising techniques informed by the review [13], their respective mechanisms of action, recommended timing of use, and the operationalization of the technique into a workable app component.

**Table 1:** Key components, mechanisms, timing of use, and operationalization in the ImpulsePal app.

Technique	Theoretical or conceptual background	Mechanism of action	Timing	Operationalization
Visuospatial Loading (eg, [32])	Elaborated Intrusion Theory of Desire [33,34]	Inhibit elaboration of craving imagery by loading the visuospatial cortex with a competing task.	This is used *in-the-moment*, when a craving occurs.	Present dynamic visual noise a visual interference pattern (such as television snow). This is triggered by pressing the emergency button and is presented in the background to the emergency button text (see in accompanying text).
Implementation Intentions (eg, [35])	Implementation Intentions [36]	Establishing goal, preempting problem situations, and making specific plans to overcome problems—specific plans (such as if-then plans) brings to mind automatically the alternative action to overcome the problem situation when it is encountered.	This technique requires users to preemptively plan for risk situations. However, the alternative response is proposed to be brought to mind *in-the-moment* when the preempted situation is encountered.	Provide option to create *if-then* plans. Prompt identification of *high-risk situations* and preemptive problem-solving, using prespecified *if* situations and *then* responses to select and save to *my plan* or to create own if-then plans.
Inhibition Training (eg, [37,38])	Associative Learning, Pavlovian conditioning, and executive response inhibition (see [39])	Improve inhibitory control, devaluing of stimuli.	The user is to engage with this training regardless of currently experiencing an eating impulse.	Present as a *Brain Training game* consisting of a stimulus-response task (go/no-go) containing images of unhealthy food/snack and drink items and neutral images (nonfood). Images of food are consistently paired with a no-go signal (appearing 100 milliseconds after the image). Neutral images are paired with go or no-go signals (50/50; appearing 100 milliseconds after the image). Feedback: For each response, a score is shown on the screen, which takes into account accuracy and speed.
Mindfulness strategies (eg, [40])	Mindfulness (see [41])	Raise awareness of the present moment by purposefully paying attention, without judgment, to the current experience that is unfolding, and observing its path without acting.	The user is to engage with this strategy *in-the-moment* when a craving is experienced. Although preemptive practice is encouraged.	Text-based steps guide the user through principles of *Urge-Surfing*. Cravings are conceptualized as being like a wave, which may build in intensity, but will eventually subside. Practice in absence of a craving is encouraged to develop this skill.
Location-specific goal primes	Goal priming (see [42])	Bringing long-term goals and goal structures.	Context-specific primes trigger goals and behavior *in-the-moment*. However, identification of risk situations/locations where a prime needs to occur requires planning and scheduling in advance.	Use of geo-caching and location services to highlight high-risk locations on a map along with specific goals for the location. Notifications are sent in the app when the user enters the location. The user is able to specify time boundaries for the notifications.

Additional components, which were identified from service user and expert consultations and additional engagement literature, were also incorporated. These included an emergency button to provide easy access to specific impulse management techniques to be used *in-the-moment*, as well as providing quick access to other techniques (see Table 1). Once the user presses the emergency button, they are presented with text congratulating them on putting their impulse on hold and prompting further action (pressing the *next* button) by saying “Now let’s see if you can take control of the situation....” This emergency button text is displayed against a background of dynamic visual noise to induce visuospatial loading, which aims to reduce craving strength by preventing the elaboration of craving imagery. Further strategies were included to enhance engagement with the intervention and effects of the behavior change techniques such as gamification (whereby users are provided with scores, which take into account both the speed and accuracy of responding to the *Brain Training* game) and personalization (in version 2 whereby the individual was able to select the food categories in the inhibition training). Participants were verbally encouraged during the baseline assessment to use the app for the first 4 weeks. However, they were allowed to use the app as much or as little as they wanted throughout the study period. After the 4 weeks, there was no further verbal encouragement given to the participants.

### Control Group (Waiting List)

Participants in the control group did not receive the ImpulsePal app during their study participation; instead, they were provided with access to the ImpulsePal app intervention after their 3-month follow-up.

### Sample Size

In line with the feasibility aims of the study, our sample size was calculated to obtain realistic estimates (and CIs) for the uptake and retention rates, as well as SDs of the primary outcome. From recent UK-based trials of interventions to support dietary change, it was estimated that 25% to 30% of those contacted would take part and of those 70% to 75% would be retained at 3 months [43,44]. A sample size of 90 would allow estimation of a retention rate of 70% to 75% with a margin of error of +/−9%. On the basis of recruiting 90 participants and assuming an uptake rate of 25%, we would need to invite 360 people, and this would yield CIs around the uptake estimate of +/−4.5%. This sample size (with a 2:1 allocation ratio) also provides an ample pool of intervention participants from whom to collect qualitative feedback. A retention rate of 70% to 75% would be large enough to allow estimation of the SD for weight loss to allow sample size calculation for a future full-scale trial [45,46].

### Blinding

Post randomization, blinding of the participant was not possible as participants were by necessity aware of whether they were receiving an app or not. In addition, the researcher was not blinded to group allocation at follow-up as interviews with the intervention group participants were conducted during the assessment visit. Blinding to group allocation during analyses was not possible either because of the uneven group sizes (2:1 allocation to intervention or control group).

### Outcomes and Measures

For this feasibility study, the main outcomes of interest were (1) uptake rate, (2) study completion rate (the proportion providing data at 3 months), and (3) the SD of weight loss at 3 months of follow-up. Other feasibility outcomes of interest were measures completion rates (the proportion of participants who completed each measure at each time point) and acceptability of the intervention and study procedures (percent satisfied with the ImpulsePal app and study procedures).

Questionnaires and study records were used to record demographic data at baseline in terms of age, gender, level of education, ethnicity, and area deprivation using the Index of Multiple Deprivation derived from postcode and national census data, which is the official measure of relative deprivation for localities in England [47]. In addition, participants reported their smoking status, any medications or diagnoses that might affect weight (such as thyroid problems), or diet (such as food allergies) and concurrent participation in other lifestyle-related weight management program at baseline, and any changes in these at 1-month and 3-month follow-up.

A full measurement schedule can be found in the Multimedia Appendix 1 (see Table S1). All measures intended for use in the full-scale trial were also taken (at baseline and follow-up, unless otherwise stated) as follows.

#### Body Measurements

Body weight in kilograms (primary outcome) was measured using calibrated scales (Seca 899 Weighing Scale). Height was measured using the Seca 213 portable stadiometer at baseline only to calculate BMI.

#### Secondary Outcomes

We measured unhealthy snack food/drink consumption using a 7-day recall 11-item food frequency questionnaire (FFQ) adapted from a questionnaire used by Churchill and Jessop [48]. This FFQ asked participants to rate how often they had eaten food from specific categories over the course of the last week. The items in the FFQ included crisps, chocolate, ice cream, chips, sweets, cakes, biscuits, pastries/sweet pies, soft drinks, low sugar/diet soft drinks, and alcoholic drinks. A 7-point response scale was presented for each item (ranging from *1=never*, *to 7=3 or more times per day*). A total FFQ index was calculated as the average of the scores. In this index, a higher score indicates more unhealthy eating as previously used by Lawrence et al [37]. In total, 2 subscales were created in the same way for the 8 snack items (FFQ Snack) and for the 3 drink items (FFQ Drink). To gather frequency data on episodes of overeating, we used 3 items from the Eating Disorder Examination Questionnaire referring to the frequency of overeating episodes and loss of control during overeating (over a period of 28 days) and the number of days in which uncontrolled overeating occurred [49].

#### App Usage

Intervention usage was measured via the app, which recorded time and date stamps for each screen visited alongside the time spent on the respective screen. For the purposes of this feasibility study, overall intervention usage is measured as the total time spent using the ImpulsePal app and the number of days the app had been accessed. However, engagement (rather than usage) with the intervention and its key components is explored in more depth in the process evaluation.

#### Feasibility of Use and Satisfaction of Users

At the 1-month assessment visit, intervention group participants were asked to complete an anonymous satisfaction questionnaire and were offered the choice of completing and returning the questionnaire at the end of the study visit (while the researcher was present) or take it home and return it by freepost envelope. The questionnaire asked about the usability of, and satisfaction with, the ImpulsePal app. For example:

How easy is ImpulsePal to understand and use?

Please indicate how satisfied you are/were with ImpulsePal.

The questionnaires used 5-point Likert response scales (1=disagree to 5=agree and 1=very dissatisfied to 5=very satisfied). In addition, an open-ended question (ie, “Is there anything we could do to improve ImpulsePal?”) was used to prompt ideas for intervention improvement.

Similar questions, with the same rating scales and return procedures described above, were asked of all participants at the 3-month visit pertaining to satisfaction with the study procedures:

The study procedures were easy to understand.

The questionnaires were easy to complete.

Is there anything we could do to improve the study?

Please indicate how satisfied you are with your research study experience.

In addition to these satisfaction questionnaires, at the 3-month follow-up (during the visit or over the phone), participants were also asked for quantitative and qualitative feedback on their trial participation experience. Questions included:

In deciding to take part in the study you were given a Participant Information Sheet. Was this helpful?

with a yes/no response,

How would you rate the amount of information that the researchers collected from you?

rating from 1-Far too much, to 5-Far too little, and

Did you have problems with your information being sent via the ImpulsePal app (intervention group only) or your weight being measured?

with a yes or no response and further comments were noted where offered.

#### Process Evaluation Measures

A mixed-methods process evaluation (which is reported elsewhere [24]) was conducted to further assess the feasibility and acceptability of the intervention in more depth, the usefulness of different intervention components, to explore mechanisms of action, and to identify ways to refine the intervention and the process measures for a full-scale trial. In brief, this incorporated (1) semistructured interviews, (2) questionnaires at baseline and follow-up to assess changes in process variables targeted by the intervention (ie, Barratt Impulsiveness Scale (BIS)-15 [50], Food Cravings Questionnaire-Trait [51]; Cognitive Restraint subscale of the Three Factor Eating Questionnaire—R18 [52]; Power of Food Scale (PFS) [53]; and a self-efficacy questionnaire constructed for this study), and (3) fidelity checks in terms of the delivery/receipt and enactment of intervention components.

### Analysis

To assess recruitment and retention, participant flow through the study was summarized using a CONSORT diagram. Recruitment and attrition rates were also summarized using descriptive statistics with 95% CIs. Completion rates are reported using frequency (N) and group percentages (%). Sample characteristics were analyzed using descriptive statistics reporting mean and SDs for continuous data and N (%) for categorical data.

Although the study was not statistically powered for between-group comparisons, we conducted exploratory analyses based on the intention-to-treat (ITT) principle where participant data were analyzed in the groups they were allocated to following randomization. Moreover, we followed a complete case principle to deal with missing outcome data (including only participants who provide data at both time points; in this study ITT and missing outcome data are considered separate issues, for a detailed discussion on the use of ITT analyses and guidance for reporting see Alshurafa et al [54]). We used analysis of covariance (ANCOVA) to compare differences in weight loss (reported as mean difference with 95% CIs) between intervention and control groups at 1 month and 3 months controlling for baseline BMI. Where baseline characteristics suggested potential differences between groups, analyses were conducted including and excluding the potential covariates to explore the sensitivity of the findings to baseline differences. We also calculated the mean changes in secondary outcomes between baseline and follow up time points for each group. Where questionnaire data were incomplete, scores were imputed using the participant’s average for the respective scale if at least 80% of the items were completed.

App usage data were analyzed using descriptive statistics reporting median and interquartile ranges, and usability and satisfaction questionnaires were analyzed using descriptive statistics, reporting means, and SDs.

## Results

### Recruitment and Retention

**Figure 1:**
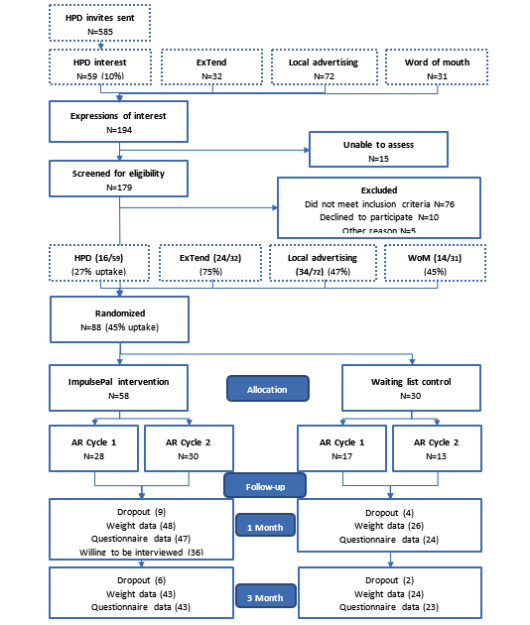
CONSORT flow diagram for the ImpulsePal feasibility study participants. One participant in the intervention group was unable to attend an assessment visit to provide weight data. AR: action research; HPD: Health Promotion Devon; WoM: word of mouth.

A total of 194 people responded to the HPD invites, local advertising, or snowballing/word-of-mouth invitations of which 93% (179/194; 95% CI 88.5% to 96.0%) were assessed for eligibility and 45% (88/194; 95% CI 38.4% to 52.4%) were eligible for inclusion and randomized into the trial between September 2015 and April 2017 (see Figure 1). Recruitment efforts stopped in April 2017 after the target number of 90 participants had been scheduled for enrolment into the study. The primary reason for exclusion was not being able to run the Android-based app (37% (66/179) of individuals assessed for eligibility). The average recruitment rate was 7.3 participants per month and was achieved with 1 researcher working on an average of 1.5 days per week during recruitment periods. Of those randomized, 84% (74/88) provided weight data at 1 month (95% CI 76.4% to 91.7%) and 76% (67/88) at 3 months (95% CI 67.2% to 85.0%; see Figure 1).

### Measures Completion, Internal Consistency, and Missing Data

The proportion of participants completing specific measures ranged from 94% (83/88) for overeating episodes to 100% (88/88) for weight at baseline, from 78% (68/88) for loss of control during overeating to 84% (74/88) for weight (and BMI) at 1 month, and from 73% (65/88) for loss of control during overeating to 76% (67/88) for weight at 3 months. Cronbach alphas for multi-item scales ranged from .64 to .96 at baseline, .62 to .96 at 1 month, and .48 to .96 at 3 months (see Multimedia Appendix 1, Table S2). Among the completed questionnaires, the most frequently missing were an item on the BIS “I plan for job security” (8% (7/88) missing) and an item assessing the participant’s confidence to successfully stick to their healthy eating goals *in the work place* (13% (11/88) missing).

### Sample Characteristics

The sample was 65% (57/88) female and 95% (81/88) white with a mean age of 46.8 years. The mean BMI was 33.3 kg/m^2^; 67% (59/88) had a BMI of 30 or higher (obese), and 26% (22/88) started the study alongside another existing weight management program. Most participants had completed professional training, undergraduate training, or a postgraduate course (71%; 60/88); were nonsmoking (91%; 80/88); and 17% (14/88) disclosed a comorbidity that might affect their weight or diet such as thyroid problems and diabetes.

There were no substantial differences between the recruited sample and the wider HPD population in terms of age and gender (Table 2), except that the recruited sample had a substantially lower BMI (mean difference −5.71 kg/m^2^; 95% CI −6.94 to −4.48). Although the BMI of the HPD participants did not differ from that of the wider HPD population, the participants who came into the study through the other recruitment routes had a substantially lower BMI than the HPD participants (mean difference −6.7 kg/m^2^), which has likely driven the difference between the recruited sample and the wider HPD population. There were no substantial differences between the participants who completed the study and those who dropped out in terms of age, gender, or BMI. Within the recruited sample, there were no substantial differences between the intervention and control groups in terms of age, gender, or other demographic variables. However, the control group was on average 5.2 kg heavier than the intervention group and had BMI scores that were 1.6 kg/m^2^ higher than the intervention group. Snacking scores from the FFQ were also slightly higher in the control group (Table 3).

**Table 2:** Characteristics of participants and the wider HPD^a^ population. The HPD invitees include those who participated in the feasibility trial as we were unable to identify them from the anonymized data provided.

Characteristics		Participants						HPD invitees	
		Non-HPD	N	HPD	N	All	N	All	N
Age (years), mean (SD)		45.6 (14.2)	71	51.8 (12.0)	16	46.8 (13.9)	87	48.0 (14.2)	585
Female, n (%)		46 (64)	72	12 (75)	16	57 (65)	88	420 (71.8)	585
Body mass index, mean (SD)		32.1 (5.4)	72	38.8 (6.1)	16	33.3 (6.1)	88	39.0 (5.4)	585
IMD^b^ score, mean (SD)		18.5 (10.7)	65	16.8 (9.3)	13	18.2 (10.4)	78	19.9 (10.0)	564
**IMD quintile, n (%)**									
	1 (least deprived)	8 (12)	65	0 (0)	13	8 (10)	78	58 (10.3)	564
	2	18 (28)	65	7 (54)	13	25 (32)	78	181 (32.1)	564
	3	17 (26)	65	2 (15)	13	19 (24)	78	172 (30.5)	564
	4	17 (26)	65	3 (23)	13	20 (26)	78	91 (16.1)	564
	5 (most deprived)	5 (8)	65	1 (8)	13	6 (8)	78	62 (11.3)	564

^a^HPD: Health Promotion Devon.

^b^IMD: Index of Multiple Deprivation.

**Table 3:** Sample characteristics at baseline.

Variable			Intervention	N	Control	N	Whole sample	N
Weight (kg), mean (SD)			93.1 (17.8)	58	98.3 (20.9)	30	94.9 (19.0)	88
Body mass index (kg/m^2^), mean (SD)			32.8 (5.6)	58	34.4 (6.9)	30	33.3 (6.1)	88
Female, n (%)			37 (64)	58	20 (67)	30	57 (65)	88
Age (years), mean (SD)			46.7 (13.6)	58	46.9 (14.8)	30	46.8 (13.9)	87
**Ethnicity, n (%)**								
	White		52 (93)	56	29 (100)	29	81 (95)	85
	Other		4 (7.1)	56	0 (0)	29	4 (4.7)	85
**Area deprivation**								
	Index of Multiple Deprivation (IMD) score, mean (SD)		17.7 (10.9)	51	19.2 (9.5)	27	18.2 (10.4)	78
	**IMD quintile, n (%)**							
		1 (least deprived)	6 (12)	51	2 (7)	27	8 (10)	78
		2	20 (39)	51	5 (19)	27	25 (32)	78
		3	10 (20)	51	9 (33)	27	19 (24)	78
		4	11 (22)	51	9 (33)	27	20 (26)	78
		5 (most deprived)	4 (8)	51	2 (7)	27	6 (8)	78
HPD referral, n (%)			10 (17)	58	6 (20)	30	16 (18)	88
Cointervention (including Orlistat), n (%)			16 (29)	56	6 (21)	28	22 (26)	84
Comorbidity, n (%)			8 (14)	56	6 (21)	28	14 (17)	84
Medication (not for weight loss but that can affect weight), n (%)			17 (30)	56	5 (18)	28	22 (26)	84
**Education, n (%)**								
	Secondary up to 16 years		7 (13)	56	3 (10)	29	10 (12)	85
	Secondary up to 18 years		5 (9)	56	3 (10)	29	8 (9)	85
	Professional training or university		39 (70)	56	21 (72)	29	60 (71)	85
	Other		5 (9)	56	2 (9)	29	7 (8)	85
**Smoking status, n (%)**								
	Never smoked		29 (52)	56	15 (54)	28	44 (52)	84
	Currently smoking		5 (9)	56	3 (10.7)	28	8 (10)	84
	Given up smoking^a^		22 (39)	56	10 (36)	28	32 (38)	84
Cognitive restraint^b^, mean (SD)			37.8 (20)	56	35.6 (18.1)	29	37.2 (19)	85
**FFQ^c^** **, mean (SD)**								
	FFQ Total		2.1 (0.4)	56	2.4 (0.8)	29	2.2 (0.6)	85
	FFQ Snack		2.1 (0.5)	56	2.3 (0.8)	29	2.2 (0.6)	85
	FFQ Drink		2.1 (0.8)	56	2.6 (1.3)	29	2.2 (1.0)	85
**Overeating, mean (SD)**								
	Overeating Frequency (number of times during 28 days)		7.6 (8.0)	55	6.6 (6.9)	28	7.2 (7.6)	83
	Loss of control (number of times during 28 days)		5.4 (7.6)	55	3.0 (4.6)	28	4.6 (6.8)	83
	Uncontrolled overeating (number of days)		5.4 (7.2)	55	4.7 (6.7)	29	5.19 (7.0)	84



**Process Questionnaires**								
	**BIS^d^** **, mean (SD)**							
		BIS—NP^e^ score	11.5 (3.6)	56	11.4 (3.2)	29	11.5 (3.4)	85
		BIS—M^f^ score	11.0 (2.8)	56	11.6 (3.8)	29	11.2 (3.2)	85
		BIS—A^g^ score	10.1 (3.1)	56	10.2 (2.5)	29	10.2 (2.9)	85
		BIS total	32.6 (7.0)	56	33.2 (7.3)	29	32.8 (7.1)	85
	PFS^h^—aggregate domains, mean (SD)		3.0 (0.8)	56	3.1 (1.0)	29	3.0 (0.8)	85
	Food Cravings Questionnaire-Trait reduced^i^, mean (SD)		59.4 (14.3)	56	60.3 (18.9)	29	59.7 (15.9)	85
	Self-efficacy^j^, mean (SD)		50.1 (14.0)	55	51.0 (21.7)	29	50.4 (16.9)	84

^a^Average 9.6 years since quit date.

^b^Cognitive Restraint scores, from 0 to 100 with higher scores indicating greater restraint.

^c^FFQ: Food Frequency Questionnaire scores, out of a maximum 7 with higher scores representing more frequent unhealthy food/snack/drink consumption.

^d^BIS: Barratt Impulsivity Scale—Short form, scores of 15 to 60 with higher scores representing higher impulsivity.

^e^NP: non-planning impulsiveness.

^f^M: motor impulsiveness.

^g^A: attentional impulsiveness.

^h^PFS: Power of Food Scale score, ranging from 1 to 5 with higher scores indicating greater susceptibility to the food environment.

^i^Food Cravings Questionnaire-Trait reduced scores ranging from 15 to 90 with higher scores indicating more thinking about food, intentions to eat, loss of control, and emotional impact on eating behavior.

^j^Self-efficacy scores ranging from 0 to 100 with higher scores representing greater confidence in ability to regulate eating habits.

### Exploratory Analyses of Weight Loss

An ITT complete case analysis (see Tables 4 and 5) showed that the intervention group lost 0.88 kg at 1 month and continued to lose weight, with an average weight loss of 1.63 kg at 3 months. The control group initially gained 0.12 kg at 1 month but then lost 0.95 kg by 3 months. Adjusting for baseline BMI, this resulted in mean differences in weight loss between groups (favoring the intervention group) of 1.03 kg at 1 month (95% CI 0.33 to 1.74), *P*=.005, and *d*=0.2 and 1.01 kg at 3 months (95% CI −0.45 to 2.47), *P*=.17 and *d*=0.2. Our sample showed a pooled SD of weight loss of 1.48 kg at 1 month and of 3.11 kg at 3 months.

### Sensitivity Analyses

Sensitivity to missing data was explored using an ITT analysis, this time dealing with missing outcome data through imputation, using the method of last observation carried forward. Adjusting for differences in baseline BMI, the pattern of weight loss remained the same, with the intervention group losing 0.91 kg more weight than the control group at 1 month, 95% CI (0.30 to 1.52), and 0.84 kg more at 3 months, 95% CI (−0.35 to 2.02). In addition, sensitivity to baseline differences in snacking behavior, cointerventions, weight-affecting medications, and ethnicity distribution was examined, and none of these factors substantially altered the pattern of the findings.

To explore the potential utility of ImpulsePal as a standalone intervention, a subgroup analysis (using an ITT with complete case analysis, see above) was conducted exploring variations in weight change alongside cointerventions. Among the control participants, those who took part in other weight management programs (23% (6/26)) lost 2.12 kg more than those who did not (85% (22/26); 95% CI 0.55 to 3.70) and 3.42 kg more at 3 months (21% (5/24) vs 79% (19/24); 95% CI −0.96 to 7.81). In the intervention group, those who engaged in cointerventions (31% (15/48)) only lost 0.49 kg more than those who used ImpulsePal as a standalone intervention (69% (33/48); 95% CI −0.35 to 1.33) and 0.96 kg at 3 months (30% (13/43) vs 69% (30/43); 95% CI −0.45 to 2.38).

### Eating Behavior

There were positive changes in nearly all measures reflecting reductions in consumption behavior and overeating, reductions in loss of control and uncontrolled eating episodes from baseline to 1 month and 3 months in both the intervention and control group, with greater reductions in the intervention group (except for drink consumption) and significantly greater reductions in frequency of loss of control during overeating and number of days of uncontrolled overeating when adjusting for baseline differences in BMI (Tables 4 and 5).

**Table 4:** Changes in the primary and secondary outcomes proposed for a full-scale trial at 1 month.

Outcome		0 to 1 month, mean (SD)	Adjusted between group mean difference^a^ (95% CI)
**Weight (kg)**			**−1.03 (−1.74 to −0.33)**
	Intervention (N=48)	−0.88 (1.34)	
	Control (N=26)	0.12 (1.73)	
**Body mass index**			**−0.36 (−0.62 to 0.11)**
	Intervention (N=48)	−0.32 (0.49)	
	Control (N=26)	0.02 (0.63)	
**FFQ^b^ total**			**−0.16 (−0.41 to 0.80)**
	Intervention (N=47)	−0.36 (0.50)	
	Control (N=24)	−0.20 (0.45)	
**FFQ snack**			**−0.19 (−0.46 to 0.08)**
	Intervention (N=47)	−0.42 (0.51)	
	Control (N=24)	−0.23 (0.60)	
**FFQ drink**			**−0.09 (−0.47 to 0.29)**
	Intervention (N=47)	−0.20 (0.79)	
	Control (N=24)	−0.11 (0.68)	
**Overeating frequency**			**−3.33 (−6.69 to 0.02)**
	Intervention (N=45)	−4.99 (7.75)	
	Control (N=24)	−1.67 (4.27)	
**Loss of control**			**−4.81 (−7.81 to −1.82)**
	Intervention (N=44)	−4.60 (7.19)	
	Control (N=24)	0.21 (2.89)	
**Uncontrolled overeating (no days)**			**−3.82 (−6.73 to −0.90)**
	Intervention (N=45)	−4.14 (6.85)	
	Control (N=24)	−0.33 (2.76)	

^a^Analysis of covariance analyses of change scores with baseline body mass index value entered into the model to adjust for baseline differences.

^b^FFQ: Food Frequency Questionnaire.

**Table 5:** Changes in the primary and secondary outcomes proposed for a full-scale trial at 3 months.

Outcome		0 to 3 months, mean (SD)	Adjusted between group mean difference^a^ (95% CI)
**Weight (kg)**			**−1.01 (−2.47 to 0.45)**
	Intervention (N=43)	−1.63 (2.1)	
	Control (N=24)	−0.95 (4.4)	
**Body mass index**			**−0.36 (−0.88 to 0.16)**
	Intervention (N=43)	−0.58 (0.76)	
	Control (N=24)	−0.35 (1.55)	
**FFQ^b^ total**			**0.07 (−0.20 to 0.33)**
	Intervention (N=43)	−0.34 (0.46)	
	Control (N=23)	−0.40 (0.58)	
**FFQ snack**			**−0.86 (−0.34 to 0.17)**
	Intervention (N=43)	−0.43 (0.46)	
	Control (N=23)	−0.34 (0.53)	
**FFQ drink**			**0.47 (0.02 to 0.91)**
	Intervention (N=43)	−0.09 (0.75)	
	Control (N=23)	−0.55 (1.01)	
**Overeating frequency**			**−2.33 (−5.79 to 1.12)**
	Intervention (N=43)	−4.87 (7.47)	
	Control (N=22)	−2.89 (4.52)	
**Loss of control**			**−3.31 (−6.65 to 0.03)**
	Intervention (N=43)	−3.76 (7.41)	
	Control (N=22)	−0.66 (3.27)	
**Uncontrolled overeating (no days)**			**−3.02 (−6.40 to 0.35)**
	Intervention (N=43)	−3.85 (7.31)	
	Control (N=22)	−1.07 (4.56)	

^a^Analysis of covariance analyses of change scores with baseline body mass index value entered into the model to adjust for baseline differences.

^b^FFQ: Food Frequency Questionnaire.

### App Use

Usable app usage statistics were available for 56 (out of 58) participants in the intervention group. The majority had seen the instructions for the app and its components (ie, brain training, urge surfing, if-then planning, emergency button, and the danger zones; Table 6), with improvements in receipt of the instructions seen after refinements to the intervention had been made (Cycle 2). The total minutes spent on the app during the first month (from first log in) ranged from 3.5 min to 446.8 min with a median usage of 38.1 min (Table 7). Of these 56 participants, 39 (70%) continued use after the first month (based on app usage statistics). Of those who did not access the app after the first month (n=17), 35% (6/17) had dropped out of the study. Usage time (total minutes or number of days) was not significantly correlated with weight loss within the intervention group either at 1 month (r=–0.16 and r=–0.01, respectively), or at 3 months (r=0.04 and r=–0.02, respectively).

**Table 6:** Delivery/receipt of intervention instructions and impulse management strategy instructions.

App component	Cycle 1 (N=26)	Cycle 2 (N=30)	Total (N=56)
First time log in, n (%)	26 (100)	28 (93)	54 (96)
App instructions, n (%)	26 (100)	29 (97)	55 (98)
Brain training, n (%)	24 (92)	30 (100)	54 (96)
Urge surfing, n (%)	22 (84)	29 (97)	51 (91)
If-then planning, n (%)	24 (92)	30 (100)	54 (96)
Emergency button, n (%)	25 (96)	30 (100)	55 (98)
Danger zones, n (%)	24 (92)	27 (90)	56 (91)

**Table 7:** App usage statistics.

Usage time period			Total minutes spent using the ImpulsePal app	Number of separate days ImpulsePal accessed
**During first month of use** **(N=56)**				
	Range		3.5 to 446.8	1 to 23
	Median (IQR^a^)		38.1 (53.7)	7.0 (5.0)
	**Excluding lost to follow-up (N=47)**			
		Range	3.48 to 446.8	1 to 23
		Median (IQR)	39.2 (54.9)	7.0 (5.0)
**During first 3 months (N=56)**				
	Range		3.5 to 1444.6	1 to 51
	Median (IQR)		46.4 (70.3)	10.0 (11.0)
	**Excluding lost to follow-up (N=41)**			
		Range	3.48 to 1444.6	1 to 51
		Median (IQR)	52.6 (96.5)	11.0 (10.5)
**Following the first month for continuing users** **(N=39)^b^**				
	Range		0.02 to 1376.10	1 to 29
	Median (IQR)		17.7 (38.7)	10 (10.3)
	**Excluding lost to follow-up (N=31)**			
		Range	0.98 to 1376.10	1 to 29
		Median (IQR)	19.1 (3.5)	5.0 (7.0)

^a^IQR: interquartile range.

^b^Use measured up until the end of the 3-month study participation.

### Feasibility of Use and Satisfaction With the ImpulsePal App

In total, 43 (74%) usable app satisfaction questionnaires were returned by the intervention group participants at 1 month. Data from these questionnaires suggested a high level of satisfaction with the intervention. In total, 98% agreed or strongly agreed that ImpulsePal was easy to understand mean 4.6 (out of 5; SD 0.6), 98% agreed or strongly agreed that ImpulsePal was easy to use mean 4.7 (SD 0.5), and 93% was satisfied or very satisfied with ImpulsePal mean 4.3 (SD 0.7). In the available app satisfaction questionnaires of Cycle 1 (n=19), the open-ended question elicited qualitative data, which suggested that (1) the Brain Training (go/no-go task) component was too lengthy (5 min) and became boring over time. Suggestions for improvement included shortening the time to complete the task and including a greater variety of images; (2) the app and strategy instructions are not always read; and (3) the Danger Zones (Global Positioning System-enabled reminders) were not accurate enough and required a better reminder system. After Cycle 2 (n=24 questionnaires), answers to the open-ended question still suggested that further improvements to the Brain Training component were required and elements of gamification were mentioned (eg, adding difficulty levels and rewards).

### Feasibility of Use and Satisfaction With the Trial Procedures

The study satisfaction questionnaires (returned by 75% (66/88) of the participants in both groups at the 3-month visit) also indicated high usability of, and satisfaction with, the trial materials and procedures. In total, 97% agreed or strongly agreed that the trial procedures were easy to understand mean 4.8 (SD 0.7) and 99% agreed or strongly agreed that the questionnaires were easy to complete mean 4.7 (SD 0.5). Finally, 96% were satisfied or very satisfied with their research study experience mean 4.7 (SD 0.5). The qualitative feedback in the open-ended questions suggested that improvements could be made to (1) the questionnaires (eg, shorter or fewer questions and the use of a Web-based form instead of pen and paper) and (2) the study visit reminder. Although this question asked participants about the study procedures, some intervention group participants were referring to the ImpulsePal app in their answer, suggesting to make ImpulsePal available on iOS or include variety in the *Brain Training* component. In addition, the brief structured interviews indicated that (1) the amount of data collected was *about right* (100%), (2) the Participant Information Sheet was helpful in their decision making about the study (85%) and some could not remember reading it (15%), (3) they did not have any issues with data being sent via the app (100%; intervention group only), and (4) they did not mind being weighed by the researcher (100%). In terms of suggested improvements, some mentioned Web-based or shorter questionnaires, better parking arrangements at the research site, and a text reminder on the day of the study visit in addition to the phone call reminder before the day.

## Discussion

**Principal Findings**

This study examined the feasibility of conducting a full-scale trial of the ImpulsePal intervention. We successfully recruited a sample of overweight adults seeking weight management support in the South West of England, suggesting that people are willing to use smartphone apps to support their weight management. This study showed acceptable uptake and retention rates and high participant satisfaction with, and use of, an intervention targeting impulsive processes to support changes in eating behavior for weight management. Moreover, this feasibility study showed high participant satisfaction with, and completion of, the trial procedures. The exploratory analysis of differences in weight loss between groups suggests that approximately 1 kg of weight loss may be achievable at the 1- and 3-month follow-up with medium and small effect sizes, respectively. It is interesting to note that app usage (total times or number of days) was not significantly associated with weight loss. This is further explored in the process evaluation [24]. On the basis of our findings, a fully powered RCT would need to recruit a total of 457 participants, assuming a pooled SD of 3.1 kg and the lower bound CI of retention (67%) to have 80% power to detect a 1.0 kg difference between groups at 3 months of follow-up at the 5% significance level. Longer term follow-up may require larger sample sizes as our data suggest that the SD for weight loss increases over time.

With regard to trial procedures, first, our uptake improved following the addition of a variety of recruitment routes (eg, local advertising and the Exeter 10,000 project newsletter). Another way in which recruitment for a full-scale trial could be further improved would be to offer ImpulsePal on devices using other operating systems in addition to Android. A third of the potentially eligible participants were eligible to take part based on age, gender, and BMI but were excluded as their devices were using iOS. Thus, there is scope for substantially extending the reach and uptake of the study. Second, retaining participants in trials of mHealth or other digital behavior change interventions is challenging [55,56] but our retention rates compare well with other digital weight management studies, which typically range from 70% to 85% at up to 3 months of follow-up [57-59]; therefore, our follow-up procedures are acceptable for use in a full-scale trial.

The pattern of weight change in this study is similar to that found in other app–based weight management interventions. One meta-analysis found that adding mHealth apps for weight management interventions significantly reduced body weight by 1.04 kg and reduced BMI by 0.43 kg/m^2^ compared with various control groups (ranging from waiting list control groups to intensive counseling [19]). However, these apps primarily focused on weight change through dietary self-monitoring, physical activity trackers, and nutritional information. To our knowledge, this is the first study to examine the potential impact of a theory- and evidence-based weight management app that explicitly targets both impulsive and reflective processes that underpin eating behavior, promoting small sustainable behavior changes without focusing on a prescribed restrictive diet.

Even if mHealth apps (including ImpulsePal) only produce 1 kg of weight loss, this is likely to be at a cost far lower than conventional interventions that deliver higher weight loss. This may result in a cost per kilogram weight loss well below that found in popular effective weight loss programs ranging from commercial programs to medications [60]. Furthermore, mHealth apps are likely to have a greater reach than face-to-face programs because of their accessibility. Nonetheless, considering 26% of our sample took part in concurrent weight management programs, it may also be interesting to investigate whether the use of ImpulsePal alongside other weight management support would result in additive effects, which may improve cost-effectiveness of existing programs [3]. In light of ongoing major cuts to public health infrastructure and services in the United Kingdom [61], including face-to-face weight management services as occurred during this study, there is a need for low-cost solutions to provide efficiencies in public health spending and mHealth may provide such solutions.

### Strengths and Limitations

The main strengths of this feasibility study were the use of rigorous methods to assess the feasibility of conducting a full-scale randomized trial of a smartphone app–based intervention using trial procedures that closely mirror those to be used in a full-scale trial, and the use of objective weight measurements to estimate SDs. However, some limitations need to be acknowledged. First, there are limitations that may have influenced the outcomes of this feasibility study. This study had a low uptake rate from the initial intended recruitment route through an existing weight management referral system (3% of those invited), which may be due to the timing of the invitation. Referrals were invited to take part in this feasibility study once they had been referred to existing local weight management groups but before commencement of their program. Therefore, this population had already been offered another service and may not have felt the need for additional support (study involvement was offered in addition to their weight management program, not as a replacement). Thus, this study failed to recruit a representative sample of the individuals referred to existing weight management interventions via primary care. However, the study successfully recruited a volunteer-based sample through additional community-based routes, which targeted overweight individuals who wanted to lose weight. However, these self-selected individuals may have been more motivated to change and do well compared with participants who are referred to weight management.

Second, because of limited resources, blinding of the researcher was not feasible. Although we used objective methods for body measurements to reduce the risk of bias, blinding of researchers collecting follow-up data would be preferable in a full-scale trial [62]. Moreover, offering the control group an alternative app with no active components would allow for blinding of the participants as well. This would minimize the potential for social desirability bias affecting self-report assessments differently between groups but would also remove any difference between the groups in motivation to stay in the trial, which was present in the current study where control participants were told they would receive the ImpulsePal at the end of their study participation (incentive). Similarly, face-to-face interviews were only conducted with intervention group participants. This qualitative evaluation may have a therapeutic effect, which may have influenced these participants over and above the ImpulsePal intervention, resulting in better outcomes in this group. The greater likelihood of a motivation to change and do well in volunteer-based samples, the potential for social desirability bias in the nonblinded assessments, and the potential therapeutic effects from the qualitative interviews may have resulted in an overestimation of the potential effect size and more favorable reports of acceptability. Moreover, satisfaction with the app was quantitatively measured using a questionnaire constructed for this study. However, a standardized satisfaction or usability questionnaire would be preferable for future evaluation.

Third, this study used a relatively short follow-up period (3 months) compared with evaluations of face-to-face weight management interventions [2]. Fourth, the minimal diversity in this sample is a limitation commonly faced by evaluations of digital weight management interventions [56]. Although the prevalence of obesity is similar for men and women, weight management trials tend to recruit samples that are on average 27% male and 73% female [63]. Our study managed to recruit a slightly higher proportion of men (35%); however, women still comprised a substantial majority. Furthermore, the majority of smartphone interventions targeting obesity have been tested in samples that were predominantly white [64] as was the sample in this study, primarily owing to its geographical location. Given that obesity and overweight differentially impact ethnic minority populations, it is important to assess the effectiveness of digital weight management interventions in diverse populations [65]. Increasing efforts to advertise the study to the male population and in additional geographical locations may provide further opportunity to extend its reach and uptake. Finally, owing to the small sample size and the fact it was a feasibility study, the comparative analysis was only exploratory, and therefore, no definitive conclusions can be drawn from differences between groups for any of the outcomes measured in this study. Therefore, a fully powered RCT is required to assess the effectiveness, and ideally cost-effectiveness, of the ImpulsePal intervention.

### Conclusions

This feasibility study demonstrated high levels of satisfaction with both the intervention and study methods. The findings suggest that an RCT is feasible, likely to recruit well, and to have good rates of follow-up. A full-scale evaluation is required to conclusively investigate the effectiveness and cost-effectiveness of ImpulsePal for people who are overweight, but initial exploratory findings are in a promising direction.

## Appendix

Multimedia Appendix 1 Supplementary file for randomised controlled feasibility trial of ImpulsePal: A smartphone app–based weight management intervention to reduce impulsive eating in overweight adults.

Multimedia Appendix 2 CONSORT-eHEALTH checklist (V 1.6.1).

## Abbreviations

ANCOVA: analysis of covariance
AR: action research
BIS: Barratt Impulsiveness Scale
BMI: body mass index
CONSORT: Consolidated Standards for Reporting Trials
FFQ: food frequency questionnaire
HPD: Health Promotion Devon
ITT: intention-to-treat
mHealth: mobile health
NHS: National Health Service
NIHR: National Institute for Health Research
PFS: Power of Food Scale
RCT: randomized controlled trial
