# Supplementary material for: Feasibility Randomized Controlled Trial of ImpulsePal: Smartphone App–Based Weight Management Intervention to Reduce Impulsive Eating in Overweight Adults
Source: JMIR Form Res. 2019 Apr 30;3(2):e11586. doi: 10.2196/11586 (PMC6658245; doi:10.2196/11586)
Supplement: Multimedia Appendix 1 [file formative_v3i2e11586_app1.pdf]

Supplementary File for Randomised Controlled Feasibility Trial of ImpulsePal: A Smartphone App-based Weight Management Intervention to Reduce Impulsive Eating in Overweight Adults.

van Beurden, S.B., Smith, J.R., Lawrence, N.S., & Abraham, C., & Greaves, C.J.

The main paper describes the protocol and findings of the ImpulsePal feasibility trial. This supplement contains further detailed information illustrating the intervention and the refinements made for the second cycle of Action Reseach, the measurement schedule, and the process evaluation. *Press Ctrl+Home to get back to this page.*

Contents

ImpulsePal Intervention Description **2**

Table S1. Measurement Schedule **4**

Table S2. Measures completion and internal consistencies **5**

References in Supplementary file **6**

ImpulsePal Intervention Description

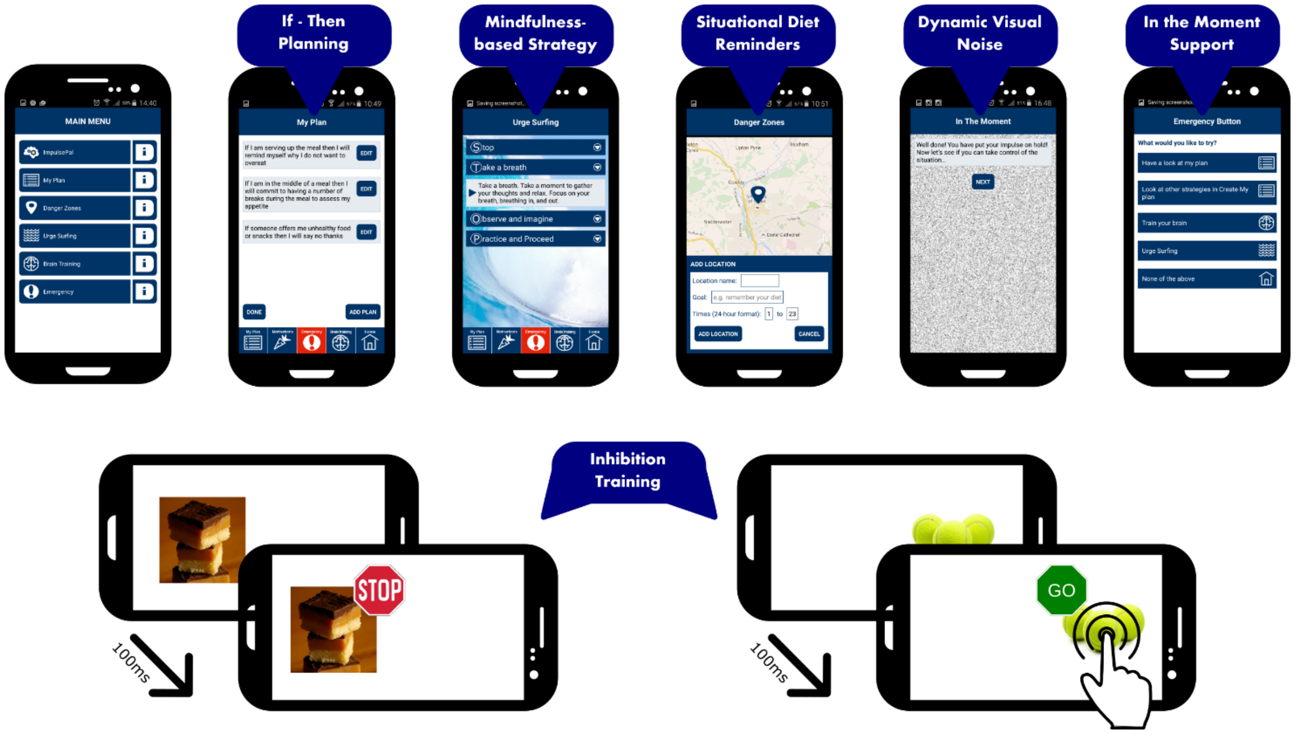

ImpulsePal is a self-delivered smartphone app that aims to help people modify or manage impulsive processes to facilitate dietary changes (i.e., reductions in snack consumption and overeating episodes). The intervention was developed using an Intervention Mapping (IM) approach [1], which includes a) a ‘needs assessment’ stage consisting of literature searches to identify relevant theory and existing evidence on impulse management and consultation with key stake holders (potential service users, experts in behaviour change and experts in app development /user engagement), b) a ‘mapping’ stage where performance objectives and determinants of change are identified along with strategies or techniques for facilitating achievement of the objectives and overcoming any barriers to change. The development was informed by dual-process theories of behaviour (e.g., Reflective Impulsive Model [2]) which highlight the importance of both impulsive and reflective processes of behaviour change. The techniques incorporated in ImpulsePal for managing impulses (via both impulsive and reflective processes) were identified in a systematic review of existing evidence [3] as being potentially promising for reducing food cravings, reducing dietary intake, or facilitating weight loss. Additional techniques were derived from consultation with service users and experts (e.g., emergency button) and literature on engagement with digital behaviour change interventions.

The following impulse management techniques are included in ImpulsePal: (1) Visuospatial loading: Operationalised as dynamic visual noise, by presenting a visual interference pattern (such as television static) to inhibit the elaboration of craving imagery [4]. (2) Implementation intentions: In the form of “if-then” planning which requires pre-emptive problem-solving for situations where the individual has identified a “high risk” of habitual or mindless unhealthy snacking as well as moments of giving in to temptations for unhealthy eating. The individual is able to select if’s and then’s from pre-populated bank of situations and responses or create their own. (3) Inhibition training: Operationalised as a Brain Training

game using a stimulus-response task (Go /no-go task) to improve inhibitory control and help people to break the learned /automated association between visual stimuli (pictures of unhealthy foods) and the "reaching out" motor cortex responses. The user is encouraged to complete this task three times per week with in-app reminders being sent out when the user has not completed the task for two consecutive days (reminders are only enabled for four weeks from download). (4) Location-specific goal primes: operationalised as self-selected textual notification messages to remind people of their own weight loss /healthy eating goals for the selected location "Danger Zones". (5) Mindfulness-based strategy: Here taught as Urge-Surfing to help people to recognise, 'hold' and process urges towards unhealthy eating when they occur. This is taught via text-based steps. Thus, ImpulsePal offers a "toolbox" of impulse management techniques which can be used as and when required by the individual.

In addition, further strategies were included to support engagement with the intervention and to reinforce the required behaviour changes. These were providing information (e.g., about how impulses may affect behaviour or about how to use specific techniques); self-monitoring of temptation resistance progress, and feedback to help people to learn from experience and build their confidence about successfully resisting temptations; promoting mental rehearsal of successful performance; and encouraging behavioural practice /repetition to increase habit and skill. Finally, the ImpulsePal app includes an Emergency Button (which is added to the phone's home screen) for when temptations or cravings are particularly strong, and the user requires in-the-moment support to help manage the situation and prevent relapse. Use of this button this leads to the above-mentioned strategies with dynamic visual noise presented straight away in the background. The user is then able to select one of the other strategies to use, or highlight that they need no further support. Fifteen minutes following pressing this button the app sends a question about temptation strength and whether or not the temptation was successfully resisted. The user is able to monitor their "temptation resistance success" in the form of a table containing information about the number of times a strategy was selected during an emergency button event and the success rate for each strategy in the previous week and overall.

Following the first cycle of action research several changes were incorporated. These included the (a) splitting the instructions over various screens, presented as a dialogue between the individual and ImpulsePal, to break up lengthy texts and promote reading of the instructions, (b) minor changes to text-based instructions to increase clarity concerning the time-boundaries for the Danger Zones to facilitate the creation of situational specific diet reminders, (c) inclusion of audio-guided urge-surfing as opposed to text-based instructions alone., (d) personalisation of the Brain Training component by incorporating various food categories that can be selected to be used for training, (e) breaking up the Brain Training component in 3 blocks to shorten the perceived time required to complete training, (f) reminder functions for the self-selected/created if-then plans to increase salience.

Table S1. Measurement Schedule

Measurement schedule

Measure	Baseline	One month	Three month
Weight (kg)	x	x	x
Height (cm)	x		
Snack consumption (FFQ)	x	x	x
Overeating episodes (EDE-Q)	x	x	x
Demographics	x	-	-
Co-intervention & Co-morbiditiy (or changes)	x	x	x
Intervention Satisfaction	-	x (intervention only)	-
Trial satisfaction	-	-	x
Process evaluation questionnaires	x	x	x
Semi-structured interview		x (intervention only)	
Restraint	x	-	-
BIS-15	x	x	x
PFS	X	x	x
FCQ-T-r	x	x	x
Self-efficacy	x	X	x
Impulse Management Strategies	-	x	x

Table S2. Measures completion and internal consistencies

	Baseline		One month		Three months	
	N	Cronbach's alpha	N	Cronbach's alpha	N	Cronbach's alpha
Weight	88 (100%)	-	74	-	67	-
BMI	88 (100%)	-	74	-	67	-
Age	87 (99%)	-	-	-	-	-
Ethnicity	85 (97%)	-	-	-	-	-
Co-intervention /or changes in	84 (95%)	-	71	-	66	-
Co-morbidity /or changes in	84 (95%)	-	71	-	66	-
Medication /or changes in	84 (95%)	-	71	-	66	-
Education level	85 (97%)	-	-	-	-	-
Smoking Status /or changes in	84 (95%)	-	71	-	66	-
Food Frequency Questionnaire	85 (97%)	0.64	71	0.62	66	0.48
Overeating Frequency	83 (94%)	-	70	--	65	-
Loss of control frequency			*69*		*64*	
No. of days			*70*		*66*	
Cognitive Restraint	85 (97%)	0.78	-	-	-	-
BIS-15	85 (97%)	0.83	71	0.83	65	0.83
PFS	85 (97%)	0.92	71	0.92	66	0.94
FCQ-T-r	85 (97%)	0.96	70	0.96	66	0.96
Self-efficacy	84 (95%)	0.85	71	0.84	66	0.90
Satisfaction App	N/A	-	43	-	-	-
Satisfaction Trial	N/A	-	-	-	66	-
Impulse Management Strategies	N/A	-	71	-	66	-

References in Supplementary file

[1] L. K. Bartholomew, G. S. Parcel, G. Kok, N. H. Gottlieb, and M. E. Fernandez, *Planning Health Promotion Programs. An Intervention Mapping Approach*, 3rd ed. San Francisco: John Wiley & Sons, Ltd, 2011.

[2] F. Strack and R. Deutsch, "Reflective and impulsive determinants of social behavior," *Personal. Soc. Psychol. Rev. Off. J. Soc. Personal. Soc. Psychol. Inc*, vol. 8, no. 3, pp. 220–247, 2004.

[3] S. B. van Beurden, C. J. Greaves, J. R. Smith, and C. Abraham, "Techniques for modifying impulsive processes associated with unhealthy eating: A systematic review," *Health Psychol.*, vol. 35, no. 8, pp. 793–806, 2016.

[4] J. May, J. Andrade, D. J. Kavanagh, and M. Hetherington, "Elaborated Intrusion Theory: A cognitive-emotional theory of food craving," *Curr. Obes. Rep.*, vol. 1, no. 2, pp. 114–121, Jun. 2012.
